# Quantitative parameters and ecological implications of a specialized tritrophic interaction involving a seed-feeding tortricid, Pseudargyrotoza conwagana, a braconid parasitoid, Bracon otiosus, and the wild privet, Ligustrum vulgare

**DOI:** 10.1093/jis/14.1.128

**Published:** 2014-09-15

**Authors:** Ángel Hernández, José Vicente Falcó

**Affiliations:** 1 Department of Agroforestry, University of Valladolid, Avenida de Madrid 44, 34004 Palencia, Spain; 2 Sustainable Forest Management Research Institute, University of Valladolid, Avenida de Madrid 44, 34004 Palencia, Spain; 3 Cavanilles Institute of Biodiversity and Evolutionary Biology, University of Valencia, C/ Catedrático José Beltrán 2, 46980 Paterna-Valencia, Spain

**Keywords:** life cycles, parasitoid sex ratio, parasitism rate, plant fitness, seed-inhabiting insects, seed infestation rate

## Abstract

Little is known about tritrophic interactions involving seed-feeding insects, parasitoid wasps, and wild fleshy fruits. Here, we examine relationships between *Pseudargyrotoza conwagana* (F.) (Lepidoptera: Tortricidae), *Bracon otiosus* Marshall (Hymenoptera: Braconidae), and the wild privet, *Ligustrum vulgare* L. (Lamiales: Oleaceae), after collecting fruits in a hedgerow habitat in northwest Spain and rearing insects indoors. No other insect species was detected in this trophic system. Each fruit contained one to four seeds, each infested fruit contained only one seed-feeding tortricid caterpillar, and each parasitized caterpillar was affected by a single braconid individual, i.e., *B. otiosus* was a solitary parasitoid. Almost half of the wild privet shrubs were infested by *P. conwagana* , and infestation ranged from 2 to 32% of fruits per infested shrub. The general effect of *P.conwagana* on wild privet dispersal can be considered low, as the overall rate of seed infestation was low (6% of seeds). The infestation rate was higher in wild privet shrubs with a larger number of seeds per fruit, and tortricid caterpillars that left the fruits successfully ate >80% of seeds. In total, the parasitism rate was moderate (25% of caterpillars), but varied considerably (0‒75%) among shrubs where *P. conwagana* infestation was detected. Parasitism only occurred in shrubs showing high infestation rates (19‒32% infested fruits), i.e., with high host densities; however, the parasitism rate was density-independent in these shrubs. The wild privets benefited from the action of *B. otiosus* in two ways: the tortricid caterpillar population was partly eliminated, and the caterpillars were prevented from eating more than one seed per fruit. The *B. otiosus* sex ratio was very balanced (1 male to 1.18 females). Winter diapause and protandry were prevalent in *B. otiosus* .

## Introduction

The proper functioning of the world’s ecosystems is critical to species survival, including humans (§ekercioğlu 2010, Tylianakis et al. 2010 ). Tritrophic interactions involving phytophagous insects, hymenopteran parasitoids, and plants are among the major terrestrial bio-tic community complexes because phytophagous insects and parasitoids represent prevalent lifestyles on earth; therefore, descriptions of these co-evolutionary triads are of immense importance to progress in knowledge of ecosystem functioning and biodiversity ( Godfray 1994 , Althoff 2003 , Santos and Quicke 2011 , Sugiura 2011 ). Most pre-dispersal seed predators are insects, and pre-dispersal seed predation, although considered to be less severe than post-dispersal seed predation, can vary considerably in space and time and have important effects on plant population dynamics and trait evolution ( Hulme 2002 , Hulme and Benkman 2002 , Fenner and Thompson 2005 , Kolb et al. 2007 ). Phytophagous insect populations may be decreased by parasitoids that cause their death before they have completed feeding, and therefore the plants may benefit indirectly because this action reduces losses in seed production ( Crabb and Pellmyr 2006 , Kevväi et al. 2006 , Abdala et al. 2010 ).

Pre-dispersal damage to ripe wild fruits by non-vertebrate agents probably occurs frequently ( Herrera 1982 ), but little is known about the natural history and effect of seed-feeding insect species associated with most fleshy-fruited plant species (e.g., Jordano et al. 2004 for the Mediterranean region). Braconid wasps are among the most commonly encountered parasitoids emerging as adults from infested fruits, but specific relationships with their hosts rarely have been established ( Wharton 2011 ). Tritrophic interactions involving seed-infesting insects, parasitoids, and wild fleshy fruit are almost unknown, with some exceptions ( Askew and Blasco-Zumeta 1997 , 2000; Satake et al. 2004 ; Crabb and Pellmyr 2006 ; Ribes and Askew 2009 ). Recently, Hernández and Falcó (2008) described for northwestern Spain a surprising tetra-trophic interaction in which adults of a *Bracon* species (Hymenoptera: Braconidae) emerged from droppings of frugivorous birds *(Turdus* blackbirds and thrushes, Turdidae) containing wild privet seeds *(Ligustrum vulgare* L., (Lamiales: Oleaceae)) (privet hereafter) infested by an unidentified tortricid caterpillar; that is, protected inside privet seeds, braconids survived after passing through the gut of birds that fed on privet fruit (see Hernández 2011 for a review on internal insect dispersal by frugivorous vertebrates). In the study by Hernández and Falcó (2008) , very few parasitoid wasps were obtained, seed infestation and parasitism rates could not be established in relationship to individual plants and fruits but only in relationship to total privet seeds contained in bird droppings, and the identity of the phytophagous caterpillar and its parasitoid could only be respectively resolved at family and genus level.

The general aim of this study is to improve our knowledge about phytophagous in-sect-parasitoid-plant interactions regarding wild fleshy fruit. Specifically, it deepens quantitative ecological understanding of the triple link among seed-feeding insects, parasitoids, and privets through new field sampling work and subsequent indoor monitoring of fruits directly collected from plants in a hedgerow habitat in northwest Spain. To achieve this objective, a more precise identification of the insects involved was accomplished, and the following aspects were analyzed: (1) the infestation rate with respect to plants, fruits and seeds, and the parasitism rate, as well as the variation between years in seed infestation and parasitism, comparing year 2007 data (this study) with 2004 data obtained by Hernández and Falcó (2008) ; (2) the correlation between infestation and the number of seeds per fruit; (3) insect survival, for the seed-feeding caterpillars and parasitoids; and (4) insect phenology, especially for the parasitoids, and the parasitoid sex ratio.

## Materials and Methods

### Study area

The study area covers 24 ha between Ruiforco and Manzaneda (30TTN93 UTM coordinates, 900 m a.s.l., León province, NW Spain) in the Supramediterranean bioclimatic stage in the Mediterranean biogeographic region, but very near the Eurosiberian region. Hot summers and cold winters characterize the area. During the privet flowering-fruiting period of 2007, the average inter-monthly mean temperature was 12.7ºC in spring (April-June), 17.2 ºC in summer (July-September), and 9.9ºC in autumn (October-November); total rainfall was 179.7 mm in spring (38 d of rain), 47.0 mm in summer (10 d), and 35.8 mm in autumn (6 d). Data were supplied by the Centro Meteorológico de Castilla y León for the La Virgen del Camino station, located near the study area at the same altitude.

Landscape is mainly composed of hedgerows that separate irrigated meadows, bordered by riparian woodland on one side and Pyrenean oak, *Quercus pyrenaica,* woods on the other. Estimated hedgerow density is 3.3 km/10 ha. Of the 27 native shrub, tree, and climber species in the hedgerows, 18 bear fleshy fruit, the most common being *Rubus* brambles (16.2% occurrences), *Rosa* roses (10.1%), privet (9.7%), and blackthorn, *Prunus spinosa,* hawthorn, *Crataegus monogyna,* and dogwood, *Cornus sanguinea* (-7-8% each) (see Hernández 2010 ). This area is of great conservation value for flora and fauna ( Hernández and Alegre 1991 ; Hernández 2007 , 2008, 2009a).

### The privet and its associated animals

The privet is a semi-evergreen shrub growing to 1.5-2.5 m (rarely up to 5 m). The flowers are grouped into more or less tight terminal panicles (racemes hereafter). The fruit is a globose berry, similar to a pea in size, black when ripe and containing one to four seeds ( López 2004 , Hernández and Falcó 2008 ). In the study area, privets bloom in late May or early June, with a peak number of flowers throughout June. The fruits begin to ripen in late August or early September, with plenty of ripe fruit in October and November, which may persist on the plant without spoiling until the end of winter (pers. obs.).

In Europe, *Ligustrum* shrub species (potentially including exotic species such as *L. lucidum* and *L. ovalifolium,* besides native *L. vulgare)* are larval foodplants for several families of Lepidoptera: Adelidae, Geometridae, Gracil-lariidae, Lycaenidae, Noctuidae, Nymphalidae, Pyralidae, Sphingidae, Yponomeutidae, and especially Tortricidae. The latter family is recorded with 16 species on *Ligustrum* of the total number of 46 moths and butterflies ( Brown et al. 2008 , Robinson et al. 2010 , Tams 2010, Unger 2011 ). The only lepidopterans recorded feeding on *L. vulgare* seeds are caterpillars of the *ade* li *d Ad-ela croesella* and the tortricid *Pseudargyrotoza conwagana.* According to direct observations of avian seed dispersers eating fruits in shrubs, common blackbirds, *Turdus merula,* make the most feeding visits to privets, whereas other species, like European robins, *Erithacus rubecula,* and some *Turdus* thrushes, make much fewer; most feeding visits by avian privet seed predators are by Eurasian bullfinches, *Pyrrhula pyrrhula* ( Snow and Snow 1988 for England; Hernández and Falcó 2008 for the study area).

### Fruit collection and monitoring of fruits and seeds in the laboratory

On 18 November 2007, 46 privet shrubs were randomly chosen in the hedgerows in the study area, and two racemes of ripe fruit were randomly chosen from each one, so that 873 fruits were collected. The two racemes were placed in a numbered bag that was then securely closed. The 46 bags containing the racemes were kept refrigerated at 8ºC from the day they were collected until 28 November 2007. On this day, the racemes from 38 shrubs (525 fruits) were used following protocol A (seed monitoring); and those from 8 shrubs (348 fruits) where at least one fruit had an exit hole, as a sign of insect infestation, were used following protocol B (fruit monitoring). In both protocols, the two racemes taken from each shrub were considered together. Other authors have followed or proposed similar protocols to achieve the emergence of parasitoids from fruits ( Askew and Blasco-Zumeta 2000 , Wharton 2011 ).

The joint objectives of protocols A and B were to identify the phytophagous and parasitoid insects at the species level and to assess overall privet infestation, parasitism and insect survival rates, general insect phenology, and sex ratio of parasitoids. The specific objectives of protocol A were to assess variation in infestation and parasitism among shrubs and monthly parasitoid emergence. The specific objectives of protocol B were to rear adult phytophagous insects and to relate variation in seed number among fruits to privet infestation and parasitism. Consequently, the main sample sizes considered in protocols A and B were *n* = 38 shrubs and *n* = 348 fruits, respectively.

### Protocol A.

Between 28 November and 2 December 2007, the number of fruits on the racemes from each shrub were counted; the fruits were examined externally (for the presence or absence of holes made by insects); the fruits were opened and the seeds examined externally (for the presence or absence of holes made by insects). The apparently intact seeds from each shrub were then placed in a small transparent plastic bag (maintaining the same numbering), which was then securely closed. All of the bags were placed in an opaque cardboard box measuring 30.5 × 18.0 × 11.5 cm (length × width × height). The box was kept in a cool laboratory room (without heating, average temperature around 18.5ºC) for 11 mo, from December 2007 to October 2008. The seeds and bags were checked at least fortnightly for the appearance of phytophagous or parasitoid insects. Room temperature was also checked on each occasion. At the end of this period, the seeds were examined externally and internally. Small pliers were used to open the apparently intact seeds. Except for fruits with exit holes, for which the number of seeds was recorded, all of the apparently intact seeds from each shrub were kept together, so the results for protocol A are mainly at shrub level. At all times, the seeds and bags were handled and checked effortlessly, therefore the results obtained for the temporal dynamics of emergence of insects are reliable.

### Protocol B.

On 28 November 2007, without removing the fruits, the racemes were placed together in a box prepared to favor pupation of phytophagous lepidopterans. The box was the same model as that used in protocol A, but an opening (24.5 × 13.5 cm) was made in the lid and covered with fine transparent glass so that the inside could be seen without touching anything. The racemes were placed on one side of the box and a bed of small pieces of crumpled, white paper on the other as a possible microhabitat for the pupation of phytophagous lepidopterans. Two small packets of silica gel were also placed in the box to absorb moisture and prevent rotting. The box was kept in the same room for the same period of time as in protocol A and checked on the same dates. At the end of this period, the inside of the box was carefully checked for insects hidden under or among the fruits and pieces of paper. The fruits and their seeds were then examined externally and internally. Small pliers were used to open the apparently intact seeds. The results of protocol B are mainly at fruit level because the racemes from all the shrubs were put together, and the number of seeds in each fruit was considered when the fruits were checked for the last time. Although the inside of the box was visible through the glass, some emerged insects could be concealed by the racemes and papers and not seen on each inspection, as verified when the box was checked for the last time, therefore giving unreliable results for the temporal dynamics of insect emergence.

### Considerations on methodology and terminology

Perforations made by caterpillars in the seed coat were circular or irregularly shaped, varied in size, and sometimes more than one was observed per seed. The exit hole made in the seed by the adult parasitoid was circular, and the whitish silken cocoon for pupation could be seen through the hole without opening the seed. Exit holes made by adult male parasitoids were considerably smaller than those made by females, in accordance with the difference in size between sexes. The seeds from which adult parasitoids emerged only had one exit hole, with no perforations made by the caterpillar. Caterpillars attacked by parasitoids usually remain paralyzed inside the first infested seed and do not leave it. Caterpillars not attacked by a parasitoid initially grow totally isolated inside a seed and perforate it later to go to another seed or leave the fruit. The number of caterpillars (and, where appropriate, parasitoids) per fruit was always one, though each caterpillar was able to feed on more than one seed in each fruit, therefore only one exit hole was observed in each fruit if the insect left it successfully.

The long ovipositor of the females made it possible to identify the sex of the parasitoids. In a few cases, it was assumed that an adult parasitoid had escaped from a bag if there was a seed with a characteristic exit hole and another similar one in the bag. “Empty” seeds are aborted seeds, partially developed but with no endosperm. “Exploitable” seeds are fully developed seeds with endosperm. “Infestation” is the action of phytophagous caterpillars.

The lepidopteran pupae we found were removed and kept in plastic vials in dry conditions. The adult wasps we found were removed and kept in plastic vials in 70% ethyl alcohol, and subsequently dried, mounted, and deposited in the ENV Entomological Collection of the Laboratorio de Entomología y Control de Plagas del Instituto Cavanilles de Biodiversidad y Biología Evolutiva, at Valencia University, Spain (collection recognized by GBIF-Spain).

The aforementioned values of autumn temperature and rainfall in the study area were estimated by considering only the first 18 d of November—on 18 November the privet fruits were collected for study. Monthly temperature in the laboratory room refers to the mean temperature estimated from the values recorded each month.

### Statistical analysis

The chi-square test ( **/** ), with Yates correction for one degree of freedom, was used to compare year 2004 data (in Hernández and Falcó 2008 ) with year 2007 data (present contribution), in reference to overall frequency of different seed types. The two-tailed Pearson product-moment correlation coefficient ( *r* ) was used to relate the number of different seed types inside the fruit, and the two-tailed unpaired *t-test* to compare the mean seed number between different fruit types, considering the fruit as the unit in both cases. The Spearman rank correlation coefficient *(r _s_ )* was used to relate orderly series of derived data (e.g., percentages) from variables whose frequency histograms clearly did not fit the bell-shaped curve of a normal distribution ( Fowler et al. 1998 ). Standard deviation (SD) was estimated as a measurement of dispersion. *P <* 0.05 was considered statistically significant.

## Results

### Phytophagous and parasitoid insect identity

**Figure 1.**
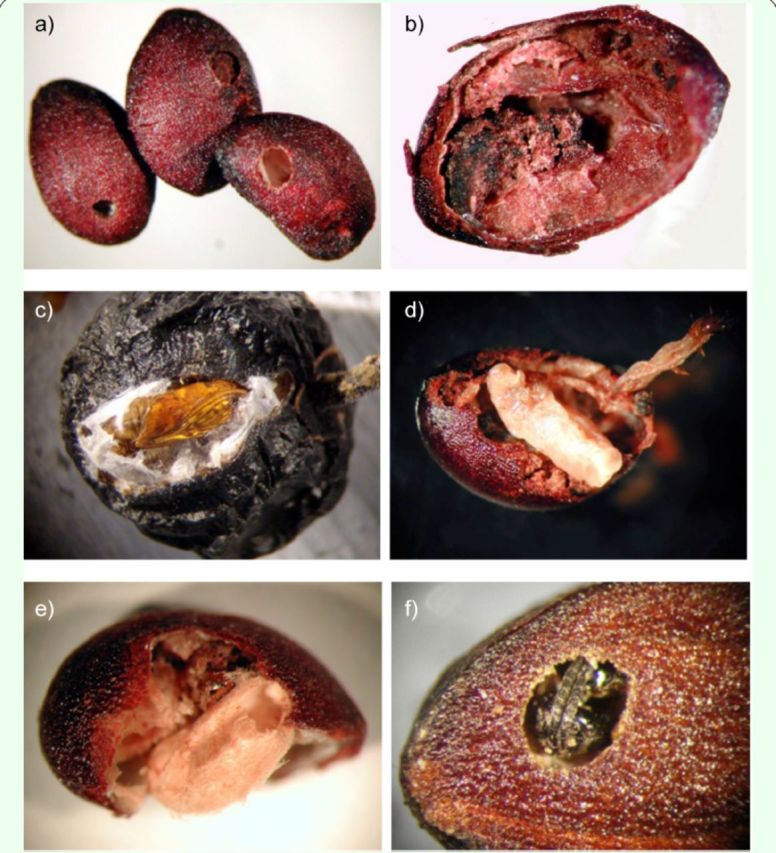
Different events in the tritrophic interaction involving the seed-feeding tortricid *Pseudargyrotoza conwagana,* the braconid parasitoid *Bracon otiosus* and the wild privet *Ligustrum vulgare.* Reference sizes: privet seeds and fruits are on average 4.5 and 7.0 mm long, respectively. **a)** Privet seeds showing exit holes of *P. conwagana* young caterpillars (left seed and central seed) and adult *B. otiosus* (right seed). **b)** Privet seed eaten by *P. conwagana* caterpillar. **c)***P. conwagana* pupa on privet fruit. **d)***B. otiosus* prepupa beside parasitized *P. conwagana* caterpillar, within privet seed. **e)***B. otiosus* silken cocoon showing exit hole, within privet seed. **f)** Adult *Bracon otiosus* just before emerging from privet seed.

No flying adult phytophagous insects were reared, but two dead pupae were found via protocol B, one hardly developed and without a cocoon, and the other partially inside the pulp of a fruit, fully developed and wrapped in a silk cocoon. The latter pupa contained a female, which allowed us to identify the species as a moth from the family Tortricidae and species *Pseudargyrotoza conwagana* (F., 1775). All emerged and captured adult parasitoids *(n =* 24 specimens, 18 from protocol A and six from protocol B), turned out to be the species *Bracon (Glabrobracon) otiosus*Marshall, 1885 (Braconidae: Braconinae), based on the description by Marshall (1885) .We assumed that parasitoids not emerging as adults *(n = 2* specimens), or ones that did emerge but could not be captured (n = 4 specimens), belonged to this species, according to the observed signs (cocoon and/or exit hole appearance). Fig. 1 shows different stages in the life cycles of *P. conwagana* and *B. otiosus* interacting with privet.

### Overall infestation, parasitism and insect survival rates; general insect phenology; sex ratio of parasitoids

**Figure 2.**
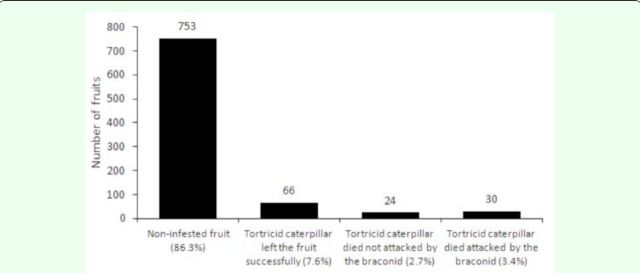
Frequency of the main types of fruits, in relationship to 873 fruits. Two racemes of fruits were collected from each of 46 privet shrubs.

The total fruits collected from privet shrubs contained 2,809 seeds, 63 (2.2%) of which were empty. Tortricid caterpillar infestation was observed in 21 (45.6%) of the 46 shrubs, and in 120 (13.7%) of the 873 fruits ( Fig. 2 ). None of the infested fruits contained empty seeds. The caterpillars had left 66 (55.0%) of the 120 infested fruits; in 24 (20.0%), they died inside the fruit without being attacked by braconid parasitoids; and in 30 (25.0%), they were attacked by braconids. Considering the fruits from which tortricid caterpillars exited, there was a significant positive correlation between the total number of seeds from each fruit (TS) and the number of seeds perforated by the caterpillar (PS) ( *r* = 0.69, df = 64, *P <* 0.01, *n =* 66 fruits; PS = 0.57TS + 0.49). In these 66 fruits, the caterpillars perforated 103 (83.1%) of 124 seeds, range 1-4 perforated seeds per fruit. Sixty-two (93.9%) of the 66 caterpillars that left the fruits had already done so in the field before the date of fruit collection (18 November). Of the 24 tortricid caterpillars found dead and not attacked by braconids, 13 (54.2%) had perforated at least one seed, and 11 (45.8%) were inside an un-perforated seed.

Of the 30 parasitoid cocoons, 27 (90.0%) were inside a seed, 2 (6.7%) were inside a seed but protruding slightly, and 1 (3.3%) was passing through two adjacent seeds. Of the 30 braconids, 28 (93.3%) emerged as adults, and 2 (6.7%) died at different stages of development inside a cocoon. Of the 28 emerged braconids, 26 (92.9%) emerged after the date when the fruits were collected (18 November); 2 (7.1%) could have emerged in the field before this date, or some time later and escaped in the laboratory room. The sex ratio of the 24 captured braconid adults was 1 male to 1.18 females.

### Variation in infestation and parasitism among shrubs; monthly braconid emergence by sex

The 525 fruits used following protocol A contained 2,183 seeds. The mean number of fruits analyzed per shrub was 36.76 ± 9.78, range 20‒62, *n* = 38 shrubs. Considering the value [total number of seeds / total number of fruits] for each shrub, a mean of 1.54 ± 0.31 seeds per fruit was obtained, range 1.1‒2.5, *n* = 38 shrubs. Empty seeds were observed in 27 (71.0%) of the 38 shrubs. The mean percentage of empty seeds per shrub was 2.21 ± 1.76%, range 0.0‒5.3%, *n* = 38 shrubs. The mean percentage of tortricid-infested fruits per infested shrub was 12.47 ± 10.51%, range 1.8‒32.4%, *n* = 13 shrubs. Considering the value [number of exploitable seeds / total number of fruits] and the percentage of infested fruits, for each infested shrub, there was a significant positive correlation between the two variables ( *r _s_* = 0.53, *P* < 0.05, *n* = 13 shrubs). Four (30.8%) of the 13 infested shrubs were affected by braconids, and the mean percentage of fruits affected by braconids in relationship to infested fruits was 46.45 ± 19.23%, range 33.3‒75.0%, *n* = 4 shrubs. These four shrubs showed the highest percentages of infested fruits (19.0‒32.4%), in contrast with the other infested shrubs (6.66 ± 5.56% infested fruits, range 1.8‒17.6%, *n* = 9 shrubs). However, there was no significant correlation between the infestation rate and the parasitism rate in braconid-infested shrubs ( *r _s_* = –0.20, *P* > 0.05, *n* = 4 shrubs).

**Figure 3.**
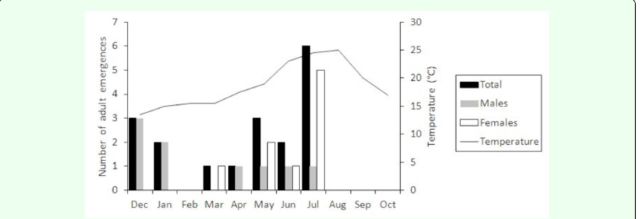
Monthly *Bracon otiosus* emergence by sex.

Between December 2007 and July 2008, 18 adult braconids emerged from the seeds with a sex ratio of 1 male to 1 female ( Fig. 3 ). Eleven (61.1%) of these specimens emerged during May-July. Six (66.7%) of nine males emerged during December-April, and eight (88.9%) of nine females emerged during May-July. Also, according to observed signs, another braconid probably emerged from the seeds in August, escaped from the corresponding bag and could not be sexed. No adult braconid emergences were detected in the last two months checked (September-October). The temperature of the laboratory room reached maximum values (19-25ºC) between May and August ( Fig. 3 ).

### Variation in seed number among fruits: its influence on infestation and parasitism

The 348 fruits used following protocol B contained 626 seeds. The mean number of seeds per fruit was 1.80 ± 0.83, range 1-4, *n =* 348 fruits. Empty seeds were observed in 12 (3.4%) of the 348 fruits; and in all cases, only one empty seed per fruit. The mean number of seeds per fruit was significantly higher in fruits with empty seeds (2.33 ± 0.65, range 1-3, *n =* 12 fruits) than in those without (1.78 ± 0.83, range 1-4, *n =* 336 fruits) *(t =* -2.28, df = 346, *P <* 0.05); but when only exploitable seeds were considered in the first type of fruit (1.33 seeds ± 0.65, range 0-2, *n =* 12 fruits), no significant difference between the two types of fruit was observed in number of seeds *(t =* 1.84, df = 346, *P >* 0.05). Of the 348 fruits, 57 (16.4%) were infested by tortricid caterpillars, and 9 (15.8%) of these fruits were affected by braconids. No significant difference was observed in the mean number of exploitable seeds per fruit between infested and noninfested fruits *(t =* -0.95, df = 346, *P >* 0.05), though it was slightly higher in the former (1.86 ± 0.77, range 1-4, *n =* 57 fruits) than in the latter (1.75 ± 0.84, range 1-4, *n =* 291 fruits). No significant difference was observed in the mean number of seeds per fruit between infested fruits with caterpillars not attacked by braconids (1.90 ± 0.75, range 1‒4, *n* = 48 fruits) and infested fruits with caterpillars attacked by braconids (1.67 ± 0.87, range 1‒3, *n* = 9 fruits) ( *t* = ‒0.82, df = 55, *P* > 0.05). There was no significant difference in the mean number of seeds per fruit between infested fruits that tortricid caterpillars had successfully left and infested fruits in which the caterpillars died without being attacked by braconids ( *t* = ‒0.15, df = 46, *P* > 0.05), though it was significantly higher in the former (1.90 ± 0.74, range 1‒4, *n* = 41 fruits) than in the latter (1.86 ± 0.90, range 1‒3, *n* = 7 fruits).

### Among-year variations in infestation and parasitism.

The relative frequency of empty seeds was significantly higher in 2004 (14 of 218 seeds, 6.4%) than in 2007 (63 of 2,809 seeds, 2.2%) ( *χ2* = 12.62, df = 1, *P* < 0.01). Considering only the total number of exploitable seeds, the relative frequency of seeds damaged by tortricid caterpillars was significantly higher in 2004 (29 of 204 seeds, 14.2%) than in 2007 (167 of 2,746 seeds, 6.1%) ( *χ2* = 18.97, df = 1, *P* < 0.01). Considering that privets have a mean number of 1.8 seeds per fruit and that caterpillars perforate most of the seeds of the fruits that they infest (data from this study), the 29 perforated seeds found in 2004 were from ≈16 fruits; therefore, the frequency of parasitism that year (5 of 16 infested fruits, 31.2%) was not significantly different to 2007 (30 of 120 infested fruits, 25.0%) ( *χ2* = 0.05, df = 1, *P* > 0.05).

## Discussion

### General features of the tritrophic interaction

In the tritrophic interaction described, only one seed-feeding insect species and one parasitoid species were detected on the privet, i.e., this is a very specialized interaction. All tortricid larvae and *Bracon* specimens found in the previous study by Hernández and Falcó (2008) on this multiple relationship show the same morphological traits and infestation/ parasitism habits, so only these three species were involved. Phytophagous insects usually feed on one or few related plant species as an adaptation to specific secondary compounds or as a strategy for escaping from natural enemies (such as parasitoids) (see Connahs et al. 2009 ). Fleshy-fruited plants normally are attacked by only one or very few seed-infesting species (e.g., Manzur and Courtney 1984 , Traveset 1993 , Traveset et al. 1995 , García et al. 2000 , Mallampalli and Isaacs 2002 , Roques and Skrzypczyńska 2003 , Satake et al. 2004 , Hernández 2009b ). Fleshy fruits often contain secondary toxic metabolites ( Herrera 1982 , 2002; Cipollini and Levey 1997 ), and, specifically, privet fruits are considered poisonous because they contain several glucosides ( Willems 1988 , Mencías and Mayero 2000 ). *Pseudargyrotoza conwagana* is present in Eurasia, and caterpillars feed on seeds of Oleaceae ( *Ligustrum* privets, *Fraxinus* ashes, *Syringa* lilacs) and Berberidaceae ( *Berberis* barberries) ( Kuznetsov 1989 , Tabrett and Hammatt 1992 , Tapper 1992a , Kelbel 1997 , Brown et al. 2008 , Robinson et al. 2010 , Tams 2011 ). In the study area, thistortricid attacks privet seeds (present study) and probably common ashes, *Fraxinus excelsior* (judging by the perforations found in some samaras, pers. obs.). No other privet seed-feeding insects were found.

In known tritrophic interactions among phytophagous insects, parasitoids, and plants, the number of parasitoid species involved is usually comparatively higher than phytophagous insect species in plants with non-fleshy fruits ( Gómez and Zamora 1994 , Edwards 1998 , Oboyski et al. 2004 , Connahs et al. 2009 , Abdala et al. 2010 , Sugiura 2011 ) and seeds of fleshy-fruited plants ( Askew and Blasco- Zumeta 1997 , 2000; Ribes and Askew 2009 ). In literature surveys by Elzinga (2005) and Santos and Quicke (2011) , averages of 2-8 parasitoid species per phytophagous species were reported. Numerous parasitoid species belonging to different hymenopteran superfamilies, such as Chalcidoidea, Ichneumonoidea, and Ceraphronoidea, affect seed-feeding insects on Mediterranean fleshy fruits *(Ephedra, Juniperus)* (3-17 parasitoid species in each plant species attack 1-2 phytophagous insect species) ( Askew and Blasco-Zumeta 1997 , 2000; Ribes and Askew 2009 ).In other cases, however, a single parasitoid species was detected ( Crabb and Pellmyr 2006 for an American fleshy-fruited yucca attacked by seed-infesting prodoxid caterpillars and parasitized by the braconid *Digonogastra* sp.).

Recorded parasitoid species attacking the tortricid *P. conwagana,* all in other European regions, are *Aprostocetus balasi* (Eulophidae: Tetrastichinae), *Scambus buolianae* (Ichneumonidae: Pimplinae), and *Clypeoplex cerophagus* (Ichneumonidae: Camplopeginae) (Graham 1987, Sedivy 2001 , Yu 2010). Most braconids are quite selective koinobiont endoparasitoids because they must circumvent the defense functions of the developing host, but Braconinae in particular (the subfamily to which *Bracon* belongs) are quite generalist idiobiont ectoparasitoids which, before oviposition, permanently paralyze their hosts, insect larvae and pupae inhabiting concealed sites such as the inside of fruits ( Askew and Shaw 1986 , Shaw and Huddleston 1991 , Falcó et al. 2006 ). Known hosts of *Bracon otiosus* in Europe are Coleoptera Curculionidae *(Anthonomus pomorum* and *Magdalis rufa),* Hymenoptera Tenthredinidae *(Nematus pumil-io),* and Lepidoptera Blastodacnidae *(Blastodacna atra)* ( Yu 2010 ), all with larvae that damage host plants feeding inside small branches or mining under bark. Our data represent the first record of *B. otiosus* from Spain and its first host-parasitoid relationship with the tortricid *P. conwagana.*

### Relationship between seed-infesting tortricids and privets

Infestation of privets by *P. conwagana* was high at population level (almost half of the shrubs were infested) but varied considerably at individual plant level (2-32% infested fruits per infested shrub). When all the analyzed seeds were considered, the infestation rate varied significantly between years (14% infested seeds in 2004, 6% infested seeds in 2007). Perhaps this variation was partly because in 2004, meteorological conditions were better for adult moth activity. The average inter-monthly mean temperature was higher in 2004 than in 2007 in spring (13.2 vs. 12.7ºC, for April-June) and summer (18.2 vs. 17.2ºC, for July-September), and total rainfall was lower in 2004 than in 2007 (146.9 vs. 226.7 mm, 43 vs. 48 d of rain, for spring and summer together) (data from the La Virgen del Camino station, near the study area). In general, moth activity increases with temperature and decreases with rainfall ( Pitcairn et al. 1990 , Holyoak et al. 1997 ).

Our results are in accordance with the great spatiotemporal variability of pre-dispersal seed predation by insects, for plants in general ( Fenner and Thompson 2005 , Kolb et al. 2007 ) and fleshy-fruited plants in particular (research in Mediterranean Spain: Askew and Blasco-Zumeta 2000 for the pteromalid wasp *Blascoa ephedrae* infesting *Ephedra distachya* and *Ephedra fragilis* seeds; Traveset 1993 for chalcidoid wasps infesting *Pistacia terebinthus* seeds). Infestation rates of *P. conwagana* on *Fraxinus* and *Syringa* species in other Eurasian regions also vary considerably; they can be very high and affect most of the harvest ( Kuznetsov 1989 , Tapper 1992b ).

The infestation rate was higher in privet shrubs with higher numbers of exploitable seeds per fruit, and tortricid caterpillars that left the fruits successfully ate >80% of exploitable seeds. Caterpillar mortality was slightly higher in fruits with fewer seeds (although not significantly, maybe due to the small sample size, *n* = 7, for infested fruits in which the caterpillars died naturally, i.e., not attacked by braconid parasitoids). Therefore, producing fruits with a large number of exploitable seeds could be disadvantageous for privets, and perhaps seed abortion, verified in most of the shrubs, was a strategy for reducing the infestation rate. In agreement with this, in 2004, with higher infestation by *P. conwagana* than in 2007, the percentage of empty privet seeds was comparatively higher (6.4 vs. 2.2%). Similarly, research by Herrera (1984) into the infestation of *Berberis vulgaris* berries by a seed-feeding tephritid fly in Spain revealed that larval mortality was higher in fruits with fewer seeds because there was less food available, that adult females selected fruits with more seeds for ovisposition, and that the seed abortion rate was lower in barberry populations where the fly was not present, coinciding with our results. Generally, seed-feeding insects select individual plants or fruits with more available seeds (e.g., Stamp 1987 , Gómez and Zamora 1994 ), although the size of the fruits and seeds can also affect fruit infestation by insects ( Gómez and Zamora 1994 , Marchand and McNeil 2006 ).

Some phenological and behavioral traits of *P. conwagana* can be deduced from our results, coinciding with those found elsewhere. Moths fly mainly from May to July; caterpillars usually feed on seeds inside fruit during September and October; full-grown larvae descend to the ground and spin a thin cocoon, and pupae usually hibernate in the upper layer of litter together with fallen leaves and fruits ( Kuznetsov 1989 , Kimber 2011 , Tams 2011 ). In agreement with this, in the study area almost 95% of the caterpillars had left the infested privet fruits by mid-November.

### Relationship between tortricids and parasitoids

The overall parasitism rate was moderately high in the two study years being considered (25-31% parasitized caterpillars), but it varied markedly among the shrubs infested by the tortricid (0-75%). In research that quantified parasitism on phytophagous insects, the rates varied considerably in different spatial and temporal scenarios for plants with non-fleshy fruits (e.g., Kevväi et al. 2006 , Abdala et al. 2010 ) and seeds of fleshy-fruited plants (e.g., Askew and Blasco-Zumeta 2000 ). In the review by Elzinga (2005) , averages of 20-70% parasitism per phytophagous insect species were found.

Parasitism only occurred in privet shrubs exhibiting high infestation rates, but, when only these shrubs were considered, the parasitism rate did not depend on the infestation rate. These results agree with several field studies showing that parasitoids aggregated to patches of higher host density, but when these patches were compared, the parasitism rate was density-independent because of such factors as mutual interference, that is, increasingly more time is spent handling and rejecting previously parasitized hosts as the density increases (see review by Heimpel and Casas 2008 ).

As well as the noticeable overall parasitism rate, adult braconid emergence was very high (> 90%), thus indicating that *P. conwagana* caterpillars were apparently high-quality hosts (as defined by Heimpel et al *.* 2003, i.e., parasitoids complete development). The *B. otiosus* sex ratio was close to equilibrium between males and females, probably because as it is a solitary parasitoid (females lay only one egg per individual host), competition for mates was population-wide. In contrast, parasitoid wasps show female-biased sex ratios if competition between sons is local, which is extreme in gregarious parasitoids (females lay multiple eggs per individual host) where there is a high probability that brother-sister matings occur; therefore, producing more sons than required to fertilize all daughters decreases the expected number of grandprogeny ( Hamilton 1967 , Godfray 1994 , Antolin 1999 ). As with almost all Hymenoptera, parasitoid wasp females can actively determine the sex of their progeny by controlling fertilization of each egg: unfertilized (haploid) eggs develop into males and fertilized (diploid) eggs into females ( Godfray 1994 ).

The main period of adult braconid emergence was May-July, coinciding with the highest temperatures and the main period of *P. conwagana* moth activity, and the males emerged before the females. In temperate climates, winter diapause and strong synchronization of their life cycle with that of their host is a strategy followed by short-lived, univoltine parasitoids specialized in temporally-restricted life stages of their hosts (see Langer and Hance 2000 , Forbes et al. 2010 ). In this scenario, early emergence is very beneficial for male parasitoids in order to arrive at mating sites as early as possible; therefore protandry (males emerge before females) is an expected phenomenon whether it be via male-biased early oviposition or via a shorter post-diapause duration of pupal stage development in males ( Forbes et al. 2010 ). A few *B. otiosus* adults emerged during winter and early spring (December-April); however, environmental conditions in the room used for rearing braconids were not the same as those in the field in the study area. For example, the indoor temperature did not go below 10ºC, but frost is very common in winter in the study area. According to Heimpel and Casas (2008) , laboratory research on parasitoids is often context-dependent and much better at telling us what parasitoids can do rather than what they actually do.

### Concluding remarks and ecological implications

The insect diversity found in our studied system was lower than expected, and the factors leading to this specialized tritrophic relationship, which could be associated with the characteristics of the insect host or the plant (see Campan and Benrey 2004 ) or with the unknown composition of the local parasitoid community, have yet to be clarified. There is a general lack of information on parasitoid communities, including braconids, in the Iberian Peninsula ( Falcó et al. 2006 ). Additional research is required on diverse abiotic and biotic factors that determine population changes at different spatial and temporal scales in tortricid-privet interactions.

The general effect of *P. conwagana* on privet dispersal estimated in the study area can be considered low. On one hand, the overall infestation percentages of germination units, i.e., seeds, were low in both years. On the other, the dispersal units, i.e., ripe fruits, were hardly affected by tortricid caterpillars on the outside, because only the seeds were eaten and not the pulp, except for the gallery exit. Therefore, frugivorous birds may have consumed infested fruits and dispersed the seeds within them that escaped insect damage. In the study area, ripe privet fruits are an important part of the diet of some seed-dispersing bird species during autumn and winter, particularly common blackbirds, *Turdus merula* (see Hernández and Falcó 2008 ).

*B. otiosus* not only benefited the privets by eliminating a significant part of the *P. conwagana* caterpillar population, but they also prevented the number of potentially viable seeds from being reduced, because almost all of the parasitized caterpillars only consumed one of the fruit seeds (fruits with parasitized caterpillars had a mean close to two seeds). Similarly, Crabb and Pellmyr (2006) found that an idiobiont braconid species of the genus *Digonogastra* had an indirect positive effect on a fleshy-fruited yucca species because it paralyzed the caterpillars of two seed-infesting moth species when they had consumed only about half as many seeds as non-parasitized caterpilllars that achieved complete development. The role of parasitoids in the natural regulation of phytophagous insect populations, with the resulting enhancement of plant fitness, also has been underlined by other authors ( Gómez and Zamora 1994 , van Loon et al. 2000 , Smallegange et al. 2008 ).

Frugivorous bird species that disperse privet seeds can also efficiently disperse braconids inhabiting privet seeds, as these insects resist passage through the entire digestive tract of birds ( Hernández and Falcó 2008 , Hernández 2011 ), thus benefiting the plant in two ways. This occurs because each individual braconid usually develops completely isolated inside a single privet seed. Dispersal of *P. conwagana* caterpillars by frugivorous birds is unlikely, because each individual caterpillar, as part of its normal development, usually feeds on more than one seed and makes large holes in the seed coat and is therefore very vulnerable to bird ingestion and digestion.
